# Effects of IKAP/hELP1 Deficiency on Gene Expression in Differentiating Neuroblastoma Cells: Implications for Familial Dysautonomia

**DOI:** 10.1371/journal.pone.0019147

**Published:** 2011-04-29

**Authors:** Rachel Cohen-Kupiec, Metsada Pasmanik-Chor, Varda Oron-Karni, Miguel Weil

**Affiliations:** 1 Department of Cell Research and Immunology, Tel Aviv University, Ramat-Aviv, Israel; 2 Bioinformatics Unit, Tel Aviv University, Ramat-Aviv, Israel; Florida State University, United States of America

## Abstract

Familial dysautonomia (FD) is a developmental neuropathy of the sensory and autonomous nervous systems. The IKBKAP gene, encoding the IKAP/hELP1 subunit of the RNA polymerase II Elongator complex is mutated in FD patients, leading to a tissue-specific mis-splicing of the gene and to the absence of the protein in neuronal tissues. To elucidate the function of IKAP/hELP1 in the development of neuronal cells, we have downregulated IKBKAP expression in SHSY5Y cells, a neuroblastoma cell line of a neural crest origin. We have previously shown that these cells exhibit abnormal cell adhesion when allowed to differentiate under defined culture conditions on laminin substratum. Here, we report results of a microarray expression analysis of IKAP/hELP1 downregulated cells that were grown on laminin under differentiation or non-differentiation growth conditions. It is shown that under non-differentiation growth conditions, IKAP/hELP1 downregulation affects genes important for early developmental stages of the nervous system, including cell signaling, cell adhesion and neural crest migration. IKAP/hELP1 downregulation during differentiation affects the expression of genes that play a role in late neuronal development, in axonal projection and synapse formation and function. We also show that IKAP/hELP1 deficiency affects the expression of genes involved in calcium metabolism before and after differentiation of the neuroblastoma cells. Hence, our data support IKAP/hELP1 importance in the development and function of neuronal cells and contribute to the understanding of the FD phenotype.

## Introduction

Familial dysautonomia (FD) is a recessive autosomal neurodegenerative disease that affects the sensory and autonomous nervous systems. Patients suffer a wide range of symptoms that include gastrointestinal dysfunction, vomiting crises, recurrent pneumonia, altered sensitivity to pain and to temperature perception, and cardiovascular instability [1]. Also, both bone mineral density and metabolism are affected in FD patients, making them prone to bone fractures, spinal cord curvature, osteoporosis and osteopenia [1], [2], [3].

The gene IKBKAP, which encodes the IKAP/hELP1 (IKAP) protein, was found to be mutated in all FD patients; the mutation in 99% of the patients is a T→C nucleotide transition in the donor splice site of intron 20. This mutation causes the skipping of exon 20 and the generation of a truncated IKAP protein because of a premature stop codon that occurs as a result of the mis-splicing event [3], [4]. An additional, rarer mutation is a G→C transversion in exon 19 that results in an arginine to proline substitution and consequently, to disruption of a consensus serine/threonine kinase phosphorylation site (RIVT→PIVT) [4], suggesting that IKAP must be phosphorylated at certain domains to be fully active.

The expression of the human IKBKAP gene is differential, being highest in the cerebellum and other neuronal tissues [4]. Also differential is the mis-splicing of exon 20 in homozygous mutation carriers (FD patients) [3], [5], where the mis-spliced variant is dominant mostly in central and peripheral neuronal tissues, leading to undetectable IKAP protein in these tissues [6].

IKAP is a subunit of the RNA Pol II Elongator complex [7], [8]. This complex was shown to be involved in varied cellular processes in yeast [9] and in mammalian cells [10]. However, recently a specific role for IKAP, not as a component of Elongator, was found in *Drosophila*, where IKAP has an RNA-dependent-RNA polymerase activity and is involved in RNAi processing [11].

The failure to generate an IKBKAP KO mouse demonstrated that the gene is essential [12] as are the IKBKAP orthologs of *C. elegans* and *Drosophila* [11]. These results and the FD phenotype in humans define FD as a developmental disease. The peripheral nervous system, mostly affected in FD, is developed from the neural crest cells, a multipotent population of migratory cells unique to the vertebrate embryo. These cells arise at the lateral edge of the neural plate and migrate throughout the embryo to give rise to a wide variety of cell types including peripheral and enteric neurons and glia, cartilage and bone, smooth muscle, and pigment cells [13].

An analysis of the role that IKAP may play in the development of peripheral neuronal cells is impaired by the fact that to date there is no good model in which the importance of IKAP in early developmental stages of peripheral neurons can be examined. We therefore used in our studies SHSY5Y, a neuroblastoma cell line of neural crest origin [14] amenable to differentiation *in vitro* [15]. We reduced the expression of IKAP in these cells and we studied the effect of IKAP downregulation on gene expression in these cells in regular growth and under differentiation.

## Results

**Figure 1:**
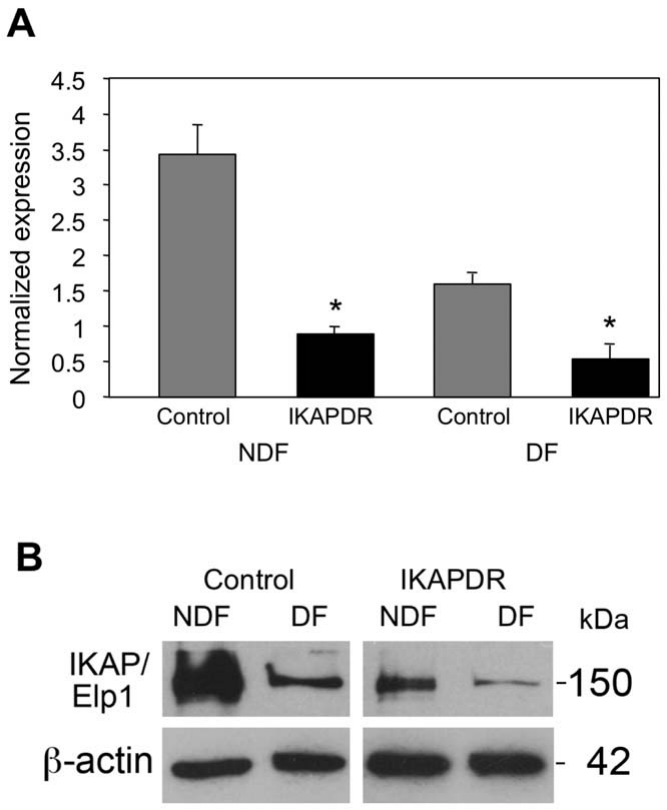
IKAP/Elp1 expression. A, qRT PCR analysis of IKBKAP gene of control (gray bars) and of IKAP-DR (Black bars) cells in non differentiation (NDF) and differentiation growth conditions (DF). Expression values of the ribosomal gene RS9 were used to normalize the expression values of IKBKAP. Each histogram represents the average of 2 different experiments each done in triplicate (a total of 6 samples) with standard errors, * = p<0.05; B, Western blot analysis of IKAP/Elp1 expression of control and IKAP-DR cells in NDF and DF growth conditions. The expression of β-actin was used as a protein loading control. Proteins molecular weights in kDa appear on the right.

To gain an understanding of the roles played by IKAP in neuronal cell growth and differentiation, we carried out a DNA microarray experiment with RNA extracted from two cell lines of SHSY5Y cells: i) IKAP-DR, in which IKAP expression was Down Regulated by an shRNA against the IKBKAP gene and ii) control cells transfected with the empty vector. The cells were grown on laminin - coated plates under two different conditions: non-differentiation; in growth medium containing 10% FC serum (NDF) or differentiation; by growth in medium with serum and the addition of retinoic acid for 5 days, followed by serum-free medium supplemented with BDNF for 3 days (DF). Figure 1A shows that IKBKAP shRNA reduced significantly the expression of the IKBKAP gene at the RNA level in non differentiation growth conditions (IKAP-DR, NDF). IKBKAP mRNA expression was further reduced upon differentiation (DF) in both control and IKAP-DR cells. These results are consistent with the microarray results, where IKBKAP was reduced in IKAP-DR cells in both NDF and DF growth conditions compared to control cells (Tables S1 and S4). Figure 1B shows IKAP protein expression of control and IKAP-DR cells at both NDF and DF growth conditions. Note that here too, IKAP expression is further reduced in both control and IKAP-DR cells in differentiation growth conditions, as reported previously [16]. The reduced expression of IKAP under differentiation is not related to the shRNA expression as it happens in control cells as well. Although reduced, IKAP protein is still present in control cells during differentiation while in IKAP-DR cells it is greatly diminished.

### Effect of IKAP depletion in non-differentiated cells (IKAP-DR-NDF versus control-NDF)

**Figure 2:**
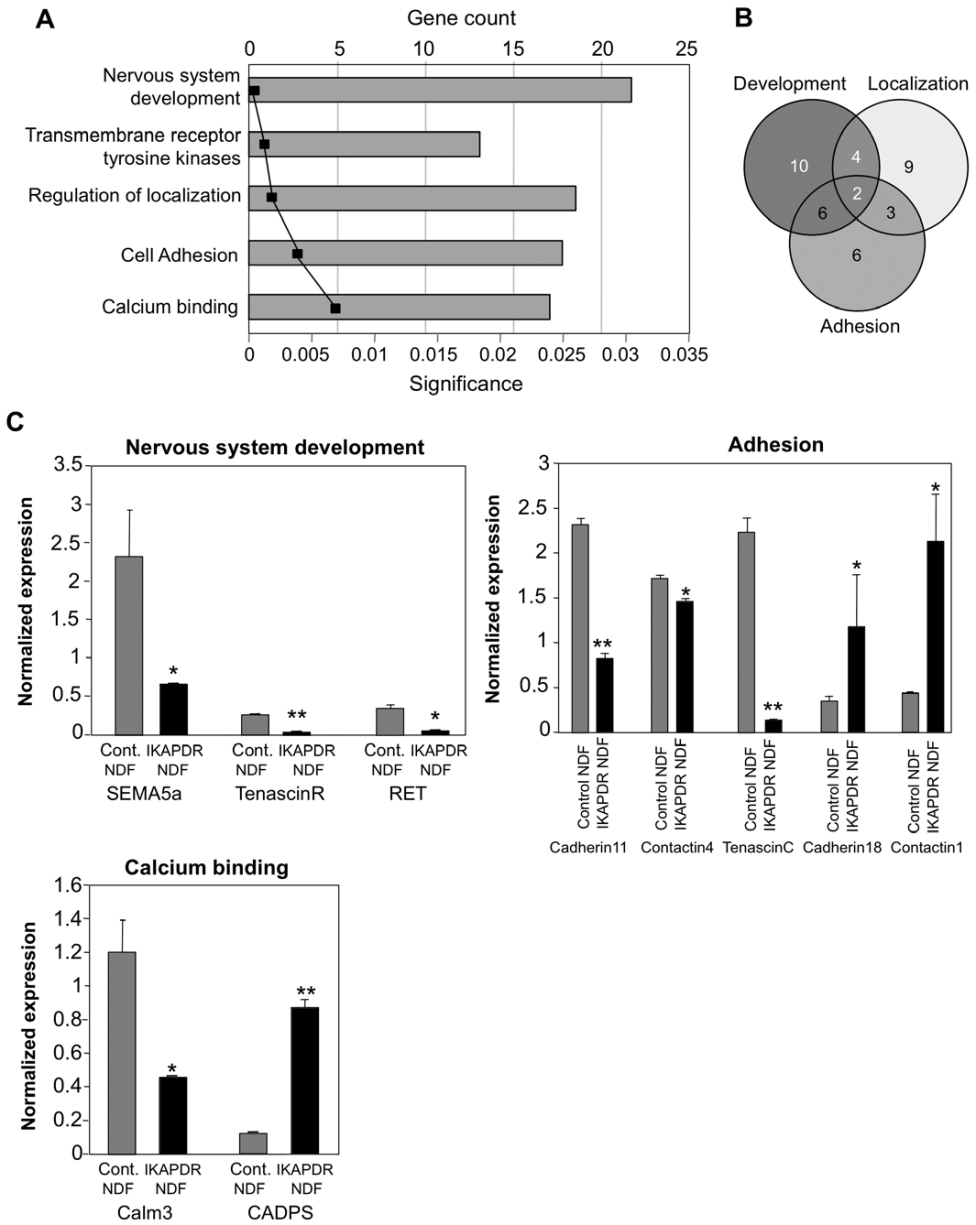
Gene expression of IKAP-DR versus control cells in non differentiation (NDF) growth conditions (1.5 fold change and *p*<0.05 under FDR (false discovery rate) adjustment criteria). A, Toppgene enriched (1.5 fold change) biological processes and molecular functions of IKAP-DR cells, diagrams show the number of genes in each group, the graph shows p-values (p<0.05 under Bonferroni correction). B, Venn diagram showing common genes among 3 of the 5 different biological processes shown in A. C, Data validation by qRT PCR of the expression levels of a few genes from the indicated groups in control (gray bars) and IKAP-DR cells (black bars) in NDF growth conditions. Each histogram represents the average of 2 different experiments each done in triplicate (a total of 6 samples) with standard errors (only >0.05 are shown). RS9 expression was used as expression normalizer; * = p<0.05, ** = p<0.01.

To examine the effect of IKAP deficiency on gene expression under non-differentiation conditions, we compared the expression data of IKAP-DR to control cells under NDF conditions. Choosing a threshold of at least 1.5 fold induction or reduction in expression, we found a total of 105 affected genes (Table S1). The expression of 78 genes was reduced while the expression of the remaining 27 genes was increased. Analysis of GO term enrichment among these genes using ToppGene [17] revealed 5 enriched biological processes ordered by statistical significance (Fig. 2A). The most significant and large category is “Nervous system development” which includes 22 genes. The other categories are: “Cell localization” (18 genes), “Enzyme-linked receptor protein signaling pathway/protein kinase signaling” (13 genes) and “Cell adhesion” (17 genes). In addition, one molecular function, “Calcium binding”, was also enriched (16 genes). Analysis of the results shows some degree of overlap between the various categories (Fig. 2B; Table S2).

Thirteen genes in the category of “Nervous system development” exhibited reduced expression in the IKAP-DR strain. These included the genes encoding semaphorins Sema3C and Sema5a, DPYSL3 (Dihydropyrimidinase, necessary for Sema3 signaling), and others such as TNR (tenascinR), TNC (tenascinC), and NAV2 (neuron navigator), all directly linked to neuronal growth. As opposed to Sema3C and Sema5a, the expression of the repulsive axon guidance semaphorin 3A (Sema3A), increased 3.6 fold in IKAP-DR cells (Table S2). Also increased was the expression of 9 genes (out of 22 in this category), including CHRNA3 (acetylcholine receptor), NTNG1 (netrin G1), ENC1 (actin binding protein involved in neural crest development), DOK4 (docking protein) and CD9 (tetraspanin). Thus, IKAP deficiency, already in non differentiation growth conditions, seems to affect nervous system development by disturbing the expression of genes involved in both pattern formation and signaling (see below).

Under non-differentiated growth conditions most of the genes related to adhesion (11 out of 17) were down-regulated in the IKAP-DR cells compared to the control (Table S2). Among these were CDH11 (cadherin11) (Fig. 2C), CADM1 (Cell adhesion molecule 1, involved in neuronal migration) and LAMA4 (laminin alpha 4, mediates cellular attachment and migration in embryonic development). On the other hand, the expression of genes like CDH18, a calcium-binding adhesion molecule that is expressed in the nervous system [18], [19] was elevated (Fig. 2C). Also, genes encoding Contactin 1(Fig. 2C) and Contactin-associated protein4 (Table S1) were expressed at higher levels. These results are consistent with the enhanced adhesion observed in IKAP-DR cells, which we have shown to be dependent on Contactin 1 [16].

Another class of genes affected by IKAP down regulation is that of transmembrane receptor protein tyrosine kinases (RTKs), which play a central role in the phosphorylation of tyrosine residues of proteins involved in signal transduction pathways [20]. Interestingly, the expression of all the nine RTKs was reduced in IKAP-DR cells, compared to control. Thus, in cells with low IKAP activity, several signal transduction pathways are down-regulated. Among the genes affected by IKAP reduction and also related to nervous system development is EGFR, an RTK from the ErbB family. Insufficient signaling of this RTK is associated with changes in gene expression, cytoskeleton arrangements and cell-cell communications affecting development of multicellular organisms [21],[22], [23]. Also reduced was RET1, which is crucial for neural crest development [24], [25], [26]. Ret1 is a gene common to several biological processes that emerge from our analysis (adhesion, calcium metabolism, development, RTK signaling, and cell localization) (Table S2). PDGFRA is also affected by IKAP reduction; this gene belongs to a family of growth factors which act as mitogens for cells of mesenchymal origin and have roles in the regulation of many biological processes including embryonic development, angiogenesis, cell proliferation and differentiation, hence contributing to the pathophysiology of some diseases [27].

Finally, another group of genes affected in the IKAP-DR strain are those related to calcium metabolism. In fact, 8 out of the 16 genes in this category are related to cell adhesion and development (Table S2), underscoring the important role that calcium metabolism plays in these processes. Several of the genes in this group are involved directly in calcium binding, such as the calmodulin-encoding gene Calm3 (reduced) and CADPS (induced), a neural/endocrine-specific cytosolic and peripheral membrane protein required for the Ca2+-regulated exocytosis of secretory vesicles [28]. Additional genes include CACNA1C (alpha1) and CACNA2D1 (alpha2/delta), encoding subunits of voltage gated calcium channels of type L [29], [30]. Interestingly, the expression of CACNA1C is reduced 2 fold while the expression of CACNA2D1 is increased 2 fold (Table S2). It is thus likely that the change in expression of these genes caused by IKAP deficiency leads to a change in the activity of these important channels, which may lead to disease [31].

We next validated the microarray results using qRT-PCR (Figure 2C). As can be seen in this figure, genes related to neuronal development: Sema5a, TNR and Ret are downregulated in IKAP-DR cells; genes related to adhesion like contactin 1 and CDH 18 are overexpressed in the IKAP-DR strains, while the expression of TNC, and CDH 11 is downregulated. The expression of calcium related genes, Calm3 is downregulated and CADPS is overexpressed.

### Differentiation affects genes related to cell division, cell-cell communication and synapse structure and function (control DF/control NDF and IKAP-DR-DF/IKAP-DR NDF)

The neural crest origin of SHSY5Y cells enables their further differentiation, manifested by the extension of neurites to generate neuronal networks. To investigate possible roles of IKAP during neuronal differentiation, we compared gene expression changes in IKAP-DR and control cells in differentiated versus non-differentiated conditions. Differentiation was achieved by treating the cells with retinoic acid followed by BDNF in serum-free medium.

**Figure 3:**
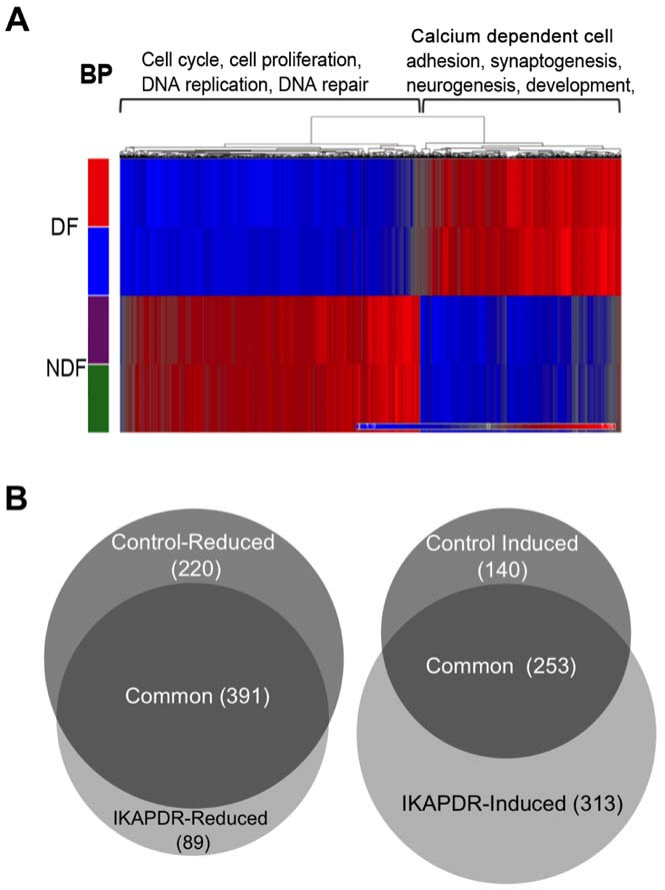
Gene expression of IKAP-DR and control cells in DF compared to NDF growth conditions. A, Clustering of increased (red) and reduced (blue) genes of control cells (each in two biological repeats) in both NDF (purple and green bars on the left) and DF (blue and red bars on the left) conditions. Representative enriched biological groups are listed at the top of the cluster (BP). B, Venn diagram (proportional) featuring common and unique reduced genes (left) and induced genes (right) between IKAP-DR and control cells in differentiation.

As expected upon differentiation, the control cells exhibited a massive reduction in the expression of genes that are involved in cell cycle (649 genes with Fold change <−1.5) (Figure 3A). These included genes involved in DNA replication, mitosis and cell division, suggesting that the cells are in a cell-cycle-arrested state. At the same time, expression of a large number of genes was increased (441 with Fold change >1.5). These genes were mainly annotated as being related to the development of the nervous system, including NCAM2, Neurexin1, doublecortin, neurofascin, synaptotagmins and synapsins to list a few, which affect both the structure and the function of the nervous system. In addition, upon differentiation of the control cells, the expression of many genes that respond to calcium (53 genes) was also increased. Several of these genes (such as Syt5, Syt9 and Syt11 encoding synaptotagmins; and a few CACNA genes encoding voltage gated calcium channels) act in nerve impulse transmission.

The expression changes observed in the IKAP-DR cells upon differentiation show a similar picture to that seen in the control cells: reduced expression of cell cycle genes in DF versus NDF on one hand and induction of genes related to neuronal development and morphogenesis on the other (data not shown). However, close inspection of the results reveals individual gene expression differences between the two cell lines (Figure 3B). The majority of the genes whose transcription was reduced upon differentiation were common to both the IKAP-DR and the control cell lines. IKAP-DR cells showed a larger number of unique genes whose expression was increased, and a smaller number of unique genes with reduced levels, compared to the control.

Among the unique genes that changed only in control cells were genes with a role during the S and M-phases of the cell cycle, including a group of histone genes involved in nucleosome and chromatin assembly (12–15 genes, respectively), all of them from a single histone cluster (located at chr6p22), whose expression was reduced only in control cells but not in IKAP-DR cells upon differentiation (Table S3A). Also, 21 genes related to calcium binding and both synapse development and signal transmission were induced only in control cells but not in IKAP-DR cells upon differentiation (Table S3B). These included a large group of genes encoding protocadherin beta 4, 6, 7, 10, 11,13, 18, and SLC25A2 which form a cluster located at chr5q31 [32], as well as calsyntenin1 [33], calsyntenin2 [34] and secretogranin II [35].

On the other hand, 24 genes whose expression was enhanced upon differentiation only in IKAP-DR cells were related to actin and cytoskeleton binding (Table S3C). These results suggest a role for IKAP in maintaining proper cytoskeleton state in the cells and also in the proper differentiation of neuronal cells, especially in the development of axons, dendrites and synapses.

### IKAP/hELP1 downregulation affects neuronal projection and synapse formation in differentiating cells (IKAP-DR-DF versus control-DF)

**Figure 4:**
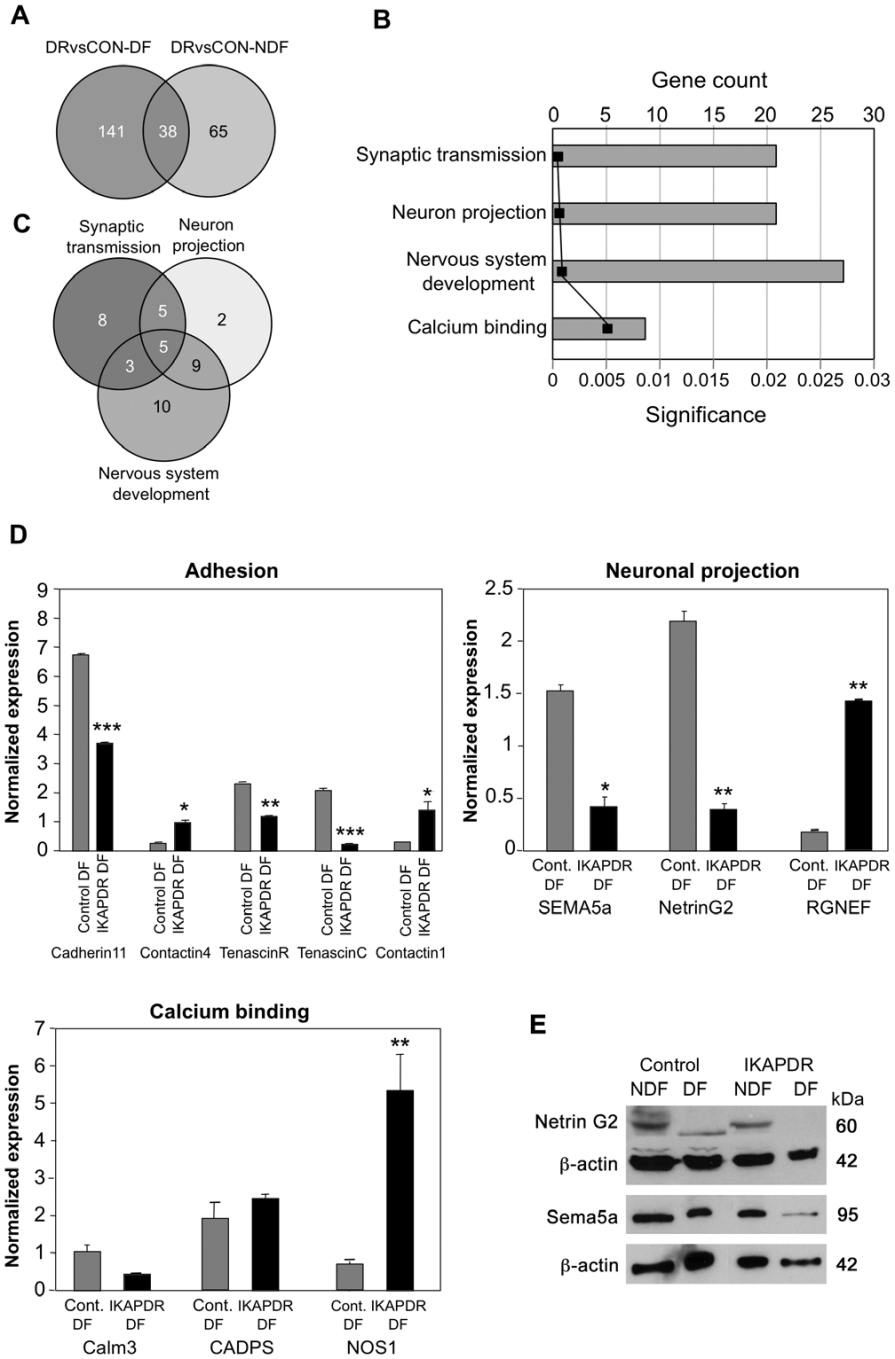
Gene expression of IKAP-DR versus control cells in differentiation (DF) growth conditions. A, Venn diagram showing the number of genes with altered expression in IKAP-DR (DR) versus control (Con), common to NDF and DF growth conditions (category A genes) and unique genes with altered expression only in DF conditions (Category B genes, left circle) or in NDF conditions (right circle). B, ToppGene enriched biological processes and molecular functions of category B genes that change in IKAP-DR cells in differentiation (p<0.05 under Bonferroni correction), diagrams show the number of genes in each group, graph shows p-values. C, Venn diagram showing common and unique genes among the 3 indicated biological processes. D, qRT PCR validation of the expression levels of a few representative genes from the indicated groups, in control (gray bars) and IKAP-DR (black bars) cells in DF. Each histogram represents the average of 2 different experiments, each in triplicate (a total of 6 samples) and standard errors (only >0.05 are shown). RS9 expression was used as expression normalizer. * = p<0.05, ** = p<0.01 *** = p<0.001. E, Western blot analyses showing the expression of NetrinG2 and Sema5a proteins in control and IKAP-DR cells in both NDF and DF growth conditions. The expression of β-actin was used as a protein loading control. Proteins molecular weights in kDa appear on the right.

By comparing the expression data of IKAP-DR cells from those of control cells in differentiated conditions, we identified 180 genes whose expression was either elevated (99) or reduced (81) in the IKAP-DR cells compared to control cells (Table S4). We divided these genes into two categories: A) Those that were already observed to be affected by IKAP deficiency in non-differentiating conditions (38 genes), and B) Genes that were not significantly affected in IKAP-DR NDF cells, but show an effect upon differentiation (141 genes) (Fig. 4A). Many of category B genes are related to development of the nervous system and specifically to neuronal projection and synapse structure and function (Figure 4B, Table S5). Some of the genes are common to these 3 biological groups (Fig. 4C).

Category A included the IKBKAP gene itself, which was reduced in non-differentiated conditions because of IKBKAP shRNA expression, and further reduced upon differentiation (Figure 1). Among the other 37 genes in category A were Sema5a, NetrinG1 and Contactin1, three genes with important roles in neurites and axonal growth. Contactin 1 was overexpressed in IKAP-DR cells in both NDF and DF growth conditions (see fig. 4D for the validation of its expression in DF, compared to other adhesion molecules). Sema5a belongs to Sema family of proteins involved in axonal guidance during neuronal development; it acts as a positive or repulsive cue for axonal guidance, depending on the nature of extracellular matrix [35]. The expression of Sema5a was reduced in IKAP-DR cells already in NDF conditions, and was further reduced in these cells upon differentiation compared to control (Fig. 2C, left panel and Fig. 4D, right panel, see also Tables S1 and S4). NetrinG1 belongs to a family of extracellular proteins that regulate the migration of neurons and axonal growth cones [36]. These proteins use bifunctional signals that are chemo-attractive for some neurons and chemo-repellent for others [37]. Interestingly, whereas the expression of NetrinG1 was induced in both NDF and DF in IKAP-DR cells, the expression of NetrinG2 (the other netrin family member) was reduced in DF (Fig. 4D, Table S4). Another category B gene, RGNEF, was induced upon differentiation (Fig. 4D, Table S4). RGNEF is a RhoA-specific guanine nucleotide exchange factor that regulates signaling pathways downstream of integrins and growth factor receptors. It functions in axonal branching, synapse formation and dendritic morphogenesis [38], [39].

Eight genes that change only in IKAP-DR but not in control cells in differentiation encode calmodulin-binding proteins, Seven of them are induced in differentiation, including CAMK4 (a calcium dependent protein kinase), SLC8A1 (a calcium transporter during excitation), GAP43 (a major component of the axonal growth cones) and MYH15 (Myosin heavy chain, an actin binding protein). Another gene worth mentioning in this category is Nos1, encoding nitric oxide synthase, also a calmodulin-binding protein whose expression was dramatically induced in IKAP-DR cells in differentiation (Fig. 4D). Nos1 plays important roles in a variety of cellular functions involving cell signaling [40]. In the nervous system, both central and peripheral, it acts as a neurotransmitter [41]. Also shown in Figure 4D are Calm3, a calmodulin binding protein that belongs to category A, and is reduced in both NDF and DF, and CADPS, that was induced in NDF (Figure 2C), but didn't change significantly in DF.

Finally, among the genes that were reduced in IKAP-DR cells compared to control in differentiation were also 9 genes encoding proteins classified as carbohydrate binding proteins (Table S4), among them APP (amyloid precursor protein) – involved in neuronal adhesion and growth, VCAN (versican), encoding an extracellular matrix protein that plays a role in cellular adhesion and development, and two UDP-N-acetyl-alpha-D-galactosamine transferases, GALNTL4 and the neuron-specific GALNT13 [42], enzymes that transfer GalNAc to mucin peptides and modify certain proteins by *O*-linked glycosylation.

We validated our results by measuring the level of Sema5a and NetrinG2, two proteins involved in axon elongation. Figure 4E shows that, in accordance to the DNA microarray and RT-PCR results, the protein levels of Sema5a and NetrinG2 are reduced in IKAP-DR compared to control upon differentiation. In addition, we noticed a difference in the apparent molecular weight of NetrinG2 in control cells upon differentiation. We hypothesize that under non differentiating conditions, this protein may be modified and hence shows slower migration in the protein gels.

## Discussion

FD is a developmental neuropathy affecting the human peripheral autonomic and sympathetic nervous systems caused by a splicing mutation in the IKBKAP gene [3]. In the mouse, the knock out of IKBKAP is lethal on early embryonic stage probably because of poor development of the embryo neuronal system and reduced development of extra embryonic constituents such as the placenta and the yolk sac [12]. Thus, IKAP is essential for the proper development of the nervous system in mammals, and especially for the development of the peripheral nervous system in humans.

All peripheral sensory and autonomic neurons (sympathetic, parasympathetic and enteric) arise from neural crest cells that migrate away from the neural tube and navigate to the location where ganglia will form. Differentiating peripheral neurons further promote axons and dendrites that must grow and connect the periphery with the spinal cord. These processes rely upon molecules that are attractive or repulsive to the migrating cells or to growth cones of growing axons [43].

SHSY5Y are undifferentiated cells of neural crest origin, but their precise developmental stage has not been characterized at the molecular level as yet. We used these cells as a model system to investigate the gene expression changes that take place upon IKAP downregulation in growth conditions that maintain regular growth and proliferation on one hand and in conditions that induce differentiation on the other hand.

Analysis of gene expression of IKAP-DR cells compared to control in non differentiation conditions showed expression differences of genes important for the early stages of nervous system development, cell adhesion, tyrosine kinases and calcium metabolism. Among the affected genes were Sema3a, Sema3C, Netrin and Ret, which are important for the migration of neural crest cells in the development of the enteric and sympathetic nervous systems (Reviewed by [43]). Snail, a transcription factor active in migrating neural crest cells [44] was also downregulated in IKAP-DR cells (Table S1). In addition, molecules of the neural extracellular matrix such as TNR and TNC (tenascinR and tenascinC, respectively), implicated in guidance of migrating neurons as well as axons during development [45] were expressed at reduced levels in IKAP-DR cells. These data point to the fact that IKAP deficiency affects the expression of molecules that serve as cues for the proper migration and guidance of neural crest cells to their destinations. This may directly affect the number of cells in the ganglia and the respective numbers of adequate projections generated from them as observed in FD [1].

A number of microarray analyses of IKAP deficient cells were previously reported; 1) Close et al. used inhibitory RNA against IKAP in HeLa cells [46]; 2) Another study, by Lee et al., [47] analyzed iPSCs generated from FD fibroblasts that were further differentiated into neural crest cells. The genes affected in these two studies did not significantly overlap our results, with the exception of two genes involved in cell motility (TNC and gelsolin that were reduced in HeLa cells and in our IKAP-DR cells - only under NDF) and SNAP91 which was reduced in the iPSCs and in our IKAP-DR cells only in differentiation. 3) Another study by Cheishvili et al., [48], which compared gene expression of healthy and FD brains, found that genes involved in myelination and development of oligodendrocytes were significantly downregulated in the FD brain. In our study we didn't see significant changes in genes that have an active role in myelination. Interestingly, however, Contactin 1, its tenascine ligands and contactin associated proteins, all involved in the formation of paranodal axo-glial junctions in myelinated peripheral neurons and in the signaling between axons and myelinating glial cells, are affected by IKAP deficiency (Figures 2 and 4 and Tables S1 and S4). The differences in gene expression patterns discussed above may be attributed to the different cell/tissue types and growth conditions used in these experiments.

Following cellular migration, neural crest cells differentiate into more specific cells, as they produce and receive local cues for proper differentiation. During differentiation cells undergo major nuclear changes that include chromatin remodeling [49]. This is accompanied by the transcriptional shut down of genes involved in cell division, and in the induction of genes acting in specific differentiation pathways. We induced neuronal differentiation and show that the expression of cell cycle and cell division genes was reduced. A few notable differences between control and IKAP-DR cells in gene regulation were evident: A cluster of histone genes at chromosome 6 was downregulated only in control cells, but not in IKAP-DR cells. One interpretation of this result is that proper regulation of this gene cluster may require the activity of IKAP, which, as part of the Elongator complex, has been shown to affect transcription [46]. The misregulation of histone genes in IKAP-DR cells could affect chromatin structure and lead to reduced expression of additional genes in differentiation. Alternatively, this result and the fact that additional DNA replication and M phase genes were affected may reflect the fact that IKAP-DR cells may have a delay in reaching the G1 arrest required for differentiation. Recently, a study by Keren et al. [50], found that FD fibroblasts are partially arrested in G1 stage of the cell cycle. These and our results suggest that IKAP deficiency affects also cell cycle regulation.

A second group of genes, which was only induced in control but not in IKAP-DR upon differentiation, is involved in calcium metabolism and included a cluster of protocadherin genes at chromosome 5, (Table S3B). Calcium is a mineral important for many cellular functions including cell adhesion and cell signaling, hence it is important for development in general, and for the development of the nervous system in particular, where it is regarded as a dominant second messenger [51]. Protocadherins are specifically active in synapse signaling [32]. Our results show changes in calcium-related genes in IKAP-DR cells in both NDF and DF growth conditions. To the best of our knowledge, this is the first report of the importance of IKAP in calcium homeostasis, which is relevant to the FD phenotype that includes calcium-related problems as spinal curvature and osteoporosis [1], [2].

On top of that, genes related to cytoskeleton and actin-binding were preferentially induced in IKAP-DR cells upon differentiation (Table S3C). Cytoskeleton problems in cells lacking IKAP have been reported [6], [52], [53]. IKAP was also found to co-localize with filamin A, an actin-binding protein, to membrane ruffles of mouse embryonic fibroblasts (MEFs) and in HeLa cells [52]. Moreover, cells lacking IKAP showed a disorganization of actin and migration problems even though there were no differences in the expression of these proteins (actin and filamin A) [52]. Here we show that IKAP deficiency induced the expression of cytoskeleton and actin-binding proteins. Actin is a constituent of axonal growth cones [54], [55], [56] and the abnormal overexpression of actin-binding proteins in differentiation may cause aberrant actin distribution both in the cytoskeleton and in growth cones, which may lead to both migration and axonal growth problems. We and others have indeed recently shown that IKAP deficient SHSY5Y cells had abnormal looking neurite projections [16].

We have also shown that upon differentiation of IKAP-DR cells, specific genes from advanced developmental stages have abnormal expression patterns as compared to control cells. These include genes involved in axon guidance, axonal growth, synapse structure and synapse function. Genes like Sema3, Ret (and other RTKs) and Snail that were affected in NDF conditions, did not change further upon differentiation. In contrast, other genes like Sema5a and NetrinG1 were affected under both NDF and DF conditions, suggesting a role for these proteins in neuronal guidance throughout neuronal migration and neurite outgrowth. These proteins indeed use bifunctional signals during development of certain neurons, and in relation to extracellular matrix [57], supporting their suggested role throughout neuronal development.

Overall, our results show that SHSY5Y cells (resembling neural crest cells), express genes required for their migration to the pertinent destinations, and upon differentiation, they express genes important for axonal growth and synapse formation. Deficiency of IKAP leads to defects at all stages of these processes. The fact that IKAP deficiency affects the expression of a large number of genes involved in the nervous system development, points strongly to the possibility that at least one of the mechanisms defective in cells lacking IKAP is transcription regulation, most probably due to lack of proper activity of the Elongator multi-protein complex of which IKAP is a central component [7], [8], [46]. A few studies have indeed shown that the elongator catalytic subunit, histone acetyl transferase, Elp3 is reduced upon IKAP deficiency, suggesting that although transcription of Elp3 is not hampered (Elp3 did not came up in our screen), still its protein level is decreased as a result of IKAP deficiency, causing reduced activity of elongator complex [46], [48], [53]. This may be added to non-transcriptional IKAP functions such as acetylation of α-tubulin which affects neuronal outgrowth and development, as demonstrated both in mouse brain and in *C. elegans* [53], [58] or acetylation of other cytoskeleton proteins, as was suggested [59].

In FD patients, IKAP depletion specifically affects the development of the sensory, the enteric and the sympathetic nervous systems. Also, FD patients exhibit severe skeleton problems that worsen with age. Our findings of the changes in the expression of genes involved in different neuronal developmental stages and in calcium metabolism in IKAP deficiency are strongly correlated with the FD phenotype.

## Materials and Methods

### Cell lines and Neuronal differentiation

SHSY5Y cells were grown in medium containing DMEM with glutamine (Gibco), 10% heat inactivated FCS (Hyclone), 2 mM sodium pyruvate (Invitrogen), and antibiotics (50 U/ml of penicillin, streptomycin and nystatin) (Biological Industries, Israel). The cell lines used in this study were described before [16]. Briefly, stable SHSY5Y cell lines were generated with IKBKAP shRNA TRCN0000037871 or with Control viruses carrying the pLKO.1-Puro control vector (Sigma) and were continually selected with 1 µg/ml puromycin (Sigma).

For differentiation, cells were plated on laminin-coated plates and grown in medium containing 10 µM retinoic acid (Peprotech Asia, Israel) for 5 days, than in serum free medium with 2 nM BDNF (Peprotech Asia, Israel) for 3 days.

### RNA extraction, microarray hybridization and data analysis

Cells were harvested by brief trypsinization and washed with PBS. Total RNA was extracted with Trisol reagent (Invitrogen), according to reagent - supplemented protocol, treated and hybridized to DNA microarrays (Affymetrix GeneChip® Human Gene 1.0 ST arrays) according to the instructions manual, as described in the Affymetrix website (http://www.affymetrix.com). We used a total of 8 chips in 2 biological duplicates. Microarray analysis was performed on CEL files using Partek® Genomics Suite TM, version 6.5 Copyright © 2010 (http://www.partek.com). Data were normalized and summarized with the robust multi-average method [60], followed by analysis of variance (ANOVA). Cluster analysis of the array was obtained by Partek® Genomics Suite TM.

Gene expression data were sorted using cutoffs of *p*<0.05 under FDR (false discovery rate) adjustment criteria of *p*<0.0002 for NDF (Table S1) and of *p*<0.0005 for DF (Table S4) [61] and fold-difference of 1.5. We used Toppgene [17] for analyses of biological and functional groups. Data were verified by Gather [58] and by David [59] - gene ontology tools. Venny was used to cross between genelists (Oliveros, J.C. 2007: http://bioinfogp.cnb.csic.es/tools/venny/index.html). All data is MIAME compliant and the raw data has been deposited in a MIAME compliant database: https://www.ncbi.nlm.nih.gov/geo/query/acc.cgi?acc=GSE26458.

### Real Time PCR

Total RNA (0.5 µg) was reverse-transcribed into complementary DNA (cDNA) with Verso 1^st^ Strand kit (ABgene, Surrey, UK) using random hexamers and according to the manufacturer's instructions. qRT-PCR was carried out using Cyber green ready mix (ABgene, Surrey, UK), and Rotor-Gene 6000 (Corbett, now Qiagen), machine and software. Primers used in these study: IKBKAP - Fwd: ATC ATC GAG CCC TGG TTT TAG, Rev: ATT GAT TCT CAG CTT TCT CAT GC; RS9- Fwd: CGG AGA CCC TTC GAG AAA TCT, Rev: GCC CAT ACT CGC CGA TCA; Contactin1 (CNTN1) - Fwd: ACC TGA ACG AAC AAC AAA ACC, Rev: ACA GGA TTT CCA AGT GCA AAA C; TenascinR (TNR) - Fwd: CCA TCT CTC CAC TCC TCA AG, Rev: CCA TCG AAG GAA AAT GAG AAG; SEMA5a -Fwd: TAG CAT GGC TGT TCT CAA GC, Rev: CAG GGG CCA ATT TCT TTA TAG; RET – Fwd: GAT CGG GAA AGT CTG TGT GG, Rev: CTG AGG TGA CCA CCC CTA GC; Cadherin11 (CDH11)-Fwd: ATC GAG AAG AGA GAG CCC AG, Rev: ACG TTG GCA TGA TAG GTC TCG; Cadherin18 (CDH18) – Fwd: GTC GCA CTC CAA TTC TGA CA, Rev: TCA CCC GTG GTA TCG TCA AT; Contactin4 (CNTN4) - Fwd: CGA GGC TTT GGT TAT GTG GTG G, Rev: CAC GCT CTC ATT CCT GAA CAC G; TenascinC (TNC) - fwd: AGG GTG GCC ACG TAC TTA CC, Rev: TGA TCT CCC AGG TTT CAA AAG C; CADPS – Fwd: TGA CTG TTG AAG AAA AGG AAC, Rev: CGA CCA AAT GGA AAG CAA TAC; CALM3 - Fwd: ATG GGA ATG GCT ACA TCA GC, Rev: GAT CAT CTC ATC CAC CTC CTC; Nos1 - Fwd: ACAGAGATTTGGACGGCAAG, Rev: TGT TGA GGA CGA CAG GCA C; NetrinG2 – Fwd : ATG CTA CGG TCA CTC CAA CC, Rev: CAG TGC TGA CCT CGC GTG; RGNEF – Fwd: TGT CAA AAG CCT GGT GGT TC, Rev: CTGCAGCAAGCAATGTCG. RS9 qRT-PCR results were used to normalize each gene expression levels in NDF or DF conditions. Reactions were performed with RNA extracted from two biological repeats in technical triplicates.

### Protein Preps and Western Blot analysis

Proteins were extracted from cell pellets using Ripa buffer (Sigma-Aldrich Corp., Israel) Protein concentrations were checked using BCA kit (Pierce Biotechnology, IL, USA). For Western analysis, 30 µg proteins were loaded on 10% acrylamide gels or 4–15% gradient gels (Bio Rad) in Tris glycin buffer. Proteins were transferred to nitrocellulose membrane and blocked in 3% low fat milk in TBST for 1 hr. Mouse momoclonal anti hIKAP (BD Biosciences, Franklin Lakes, New Jersey,USA), or mouse anti β-actin ((Sigma-Aldrich Corp., Israel) (both at 1∶1000) were applied for 1 hr at room temperature. Mouse polyclonal anti human netrin G2 and anti human Sema5a antibodies (Novus biologicals; USA) were used at 1∶1000 for over night at 4°C. Secondary antibody was Donkey anti-mouse –HRP conjugated (at 1∶6000) (Jackson ImmunoResearch laboratories, West Grove, PA, USA). For ECL, Super signal kit (Pierce Biotechnology, IL, USA) was used.

## Supporting Information

Table S1 A list of genes with reduced or increased expression in IKAP-DR versus control cells in non differentiation growth conditions (NDF). Gene expression data were sorted using cutoff of *p*<0.05 under FDR (false discovery rate) adjustment criteria of *p*<0.0002. Exceptions are NTC and SEMA3a which are significant but failed the FDR cut off (*p* = 0.001769 and 0.00044, respectively).
(XLS)

Table S2 Genes that their expression was changed in IKAP-DR versus control cells under NDF growth conditions which are common to a few of the enriched biological processes. Genes common to 4 or more biological processes are colored.
(XLS)

Table S3 A, a list of the histone genes (clustered at chromosome 6) that were reduced only in control cells under differentiation. Also indicated is the fold difference in expression of each gene in differentiation compared to undifferentiation growth conditions. B, a list of calcium-ion binding genes induced only in control cells. Also indicated at the bottom of the figure is a group of genes clustered in chr5q31, that include 8 protocadherins and the solute carrier gene SLC25 along with their fold difference of expression in DF versus NDF growth conditions. C, a list of cytoskeleton-binding proteins induced only in IKAP-DR cells, this group includes 18 genes which are actin binders.
(XLS)

Table S4 A list of genes with reduced or increased expression in IKAP-DR versus control cells in differentiation growth conditions (DF). Gene expression data were sorted using cutoffs of *p*<0.05 under FDR (false discovery rate) adjustment criteria of *p*<0.0005.
(XLS)

Table S5 A, Synapse and synaptic-transmission genes enriched in IKAP-DR cells compared to control in differentiation growth conditions (DF). B, Neuron projection genes enriched in IKAP-DR cells in DF. C, Nervous system development genes enriched in IKAP-DR in DF.
(XLS)
